# Editorial: Breast cancer imaging: clinical translation of novel methods

**DOI:** 10.3389/fonc.2025.1581169

**Published:** 2025-03-20

**Authors:** Sai Man Cheung, Simone Palma, Luca Nicosia, Jiabao He

**Affiliations:** ^1^ Newcastle Magnetic Resonance Centre, Translational and Clinical Research Institute, Faculty of Medical Sciences, Newcastle University, Newcastle upon Tyne, United Kingdom; ^2^ Department of Diagnostic Imaging, Oncological Radiotherapy and Hematology, Fondazione Policlinico Universitario Agostino Gemelli IRCCS, Rome, Italy; ^3^ Breast Imaging Division, Radiology Department, IEO European Institute of Oncology IRCCS, Milan, Italy

**Keywords:** breast cancer imaging, clinical translation, MRI, ultrasound, early diagnosis, treatment monitoring

Breast cancer has become the most common cancer in women, and the incidence rate has increased by 6% in the last decade (1) with a projected increase of 2% between 2024 and 2035 (2). In the EU, women over 50 years of age receive regular radiological screening (3), while younger women at high risk of developing breast cancer receive annual surveillance (4). However, current radiological approaches are suboptimal and suffer from high false positive and negative rates (5), leading to overtreatment and late detection (6, 7). There is an urgent unmet clinical need for novel radiological methods to facilitate accurate early detection and treatment monitoring of breast cancer. Current radiological methods for breast cancer diagnosis and treatment monitoring are primarily mammography, ultrasound and MRI (8). Mammography is primarily sensitive to the presence of microcalcifications in the tumor, ultrasound is sensitive to solid masses in the tumor versus fluid-filled lesions, and MRI is sensitive to the presence of abnormal vasculature in the tumor (8). However, there have been major advances in medical imaging in recent years, ranging from novel ultrasound devices and algorithms to functional and metabolic MRI. These innovations not only have the potential to improve the accuracy of diagnosis but may also provide critical information for treatment planning that was previously unavailable from radiological examination, leading to a change in healthcare pathway. We, therefore, would like to highlight recent developments in breast imaging methods (6 articles on ultrasound techniques and 3 on MRI techniques) with this Research Topic to facilitate clinical translation.

Conventional B-mode ultrasound reconstructs breast anatomy from the reflection of high-frequency acoustic waves at the interface of tissue boundaries, offering a tool with the advantages of low cost, safety, speed, wide accessibility and high sensitivity in dense breast, and the disadvantages of limited image contrast and operator dependence (9). With a contrast agent to highlight blood flow, Li et al. explored the relationship of perfusion characteristics with molecular subtypes, and identified heterogeneous enhancement, perfusion defects and peripheral radial vessels for grade III tumors, perfusion defects and clear edges after enhancement for human epidermal growth factor receptor 2 (HER-2) and triple-negative breast cancer (TNBC), and peak enhancement and wash-in perfusion for Luminal A and Luminal B differentiation. Using shear wave elastography to reveal mechanical properties and super microvascular imaging to outline microcirculation, Wang et al. investigated the inclusion of quantitative tumor properties in the breast-imaging reporting and data system (BI-RADS), and achieved higher sensitivity (+1.5%), specificity (+16.0%) and accuracy (+13.2%) than the conventional classification. Using strain elastography and an automated breast volume scanner to form a comprehensive picture, Shiyan et al. studied the risk of malignancy in hypoechoic lesions using a radiomics approach and constructed a nomogram using multivariate logistic regression with a larger area under the curve (AUC) in receiver operating characteristic (ROC) than the BI-RADS and clinical risk factors model alone. Leveraging artificial intelligence to accelerate workflow and reduce operator dependence (10), Qiu et al. trained a breast lesion classification algorithm using dynamic ultrasound videos from two hospitals and demonstrated a higher consistency closer to the experienced clinicians (Kappa: 0.82) than the junior clinicians (Kappa: 0.60) for diagnostic efficiency. Combining automated breast volume scanners and artificial intelligence to extract clinically relevant features, Li et al. estimated the probability of malignancy for ambiguous BI-RADS 4 lesions using radiomics features and showed an AUC of 0.949, a sensitivity of 82.14% and specificity of 95.56%. However, with RECIST criteria for treatment monitoring abandoned in many centers, Zhang et al. attempted to use an artificial intelligence algorithm trained on patients undergoing neoadjuvant chemotherapy (NACT) for pathological complete response identification but failed to show any significant improvement in AUC over manual and conventional approaches.

Conventional dynamic contrast-enhanced (DCE) MRI highlights the vascular abnormalities associated with angiogenesis in breast tumors using a paramagnetic contrast agent, offering a tool with the advantages of high resolution, high sensitivity, and good contrast in dense breast, and the disadvantages of limited image contrast, high cost, and potential adverse reactions to contrast agent (11). Using diffusion MRI for tissue microstructure profiling and an extension of intravoxel incoherent motion (IVIM) for concurrent microcirculation estimation without a contrast agent, Cheung et al. investigated tumor cellular microstructure and perfusion using a Bayesian algorithm for noise reduction as early response markers for NACT and found a decrease in perfusion fraction in good responders against an increase in poor responders after 1 cycle of NACT. However, with mathematical modeling of DCE MRI to derive quantitative perfusion characteristics, Almutlaq et al. explored the discrepancies in tumor perfusion quantified by IVIM and DCE MRI and illustrated the discordance between the two imaging techniques on perfusion but at the same time the concordance between interstitial and extracellular volume fractions against water diffusion. Using 2D, 3D and hotspot region-of-interest analysis approaches in conjunction with quantitative metrics of apparent diffusion coefficient to reduce operator dependency, Biswas et al. derived the optimal cutoffs with 2 and 4 diffusion weightings using two larger clinical trials [ECOG-ACRIN A6702 (12) and EUSOBI (13)] and recommended the hotspot approach with 2 diffusion weightings for differentiating between malignant and benign lesions.

We thank all the contributors for their excellent research work that advances medical imaging for the diagnosis and prognosis of breast cancer. Together, we can help humankind identify breast cancer early and treat it more gently.
